# The Introduction of a New Diagnostic Tool in Forensic Pathology: LiDAR Sensor for 3D Autopsy Documentation

**DOI:** 10.3390/bios12020132

**Published:** 2022-02-19

**Authors:** Aniello Maiese, Alice Chiara Manetti, Costantino Ciallella, Vittorio Fineschi

**Affiliations:** 1 Department of Surgical Pathology, Medical, Molecular and Critical Area, Institute of Legal Medicine, University of Pisa, 56126 Pisa, Italy; aniello.maiese@unipi.it (A.M.); a.manetti3@studenti.unipi.it (A.C.M.); 2 Department of Anatomical, Histological, Forensic and Orthopedic Science, Sapienza University of Rome, 00161 Rome, Italy; costantino.ciallella@uniroma1.it

**Keywords:** autopsy record, three-dimensional model, LiDAR sensor

## Abstract

Autopsy is a complex and unrepeatable procedure. It is essential to have the possibility of reviewing the autoptic findings, especially when it is done for medico-legal purposes. Traditional photography is not always adequate to record forensic practice since two-dimensional images could lead to distortion and misinterpretation. Three-dimensional (3D) reconstructions of autoptic findings could be a new way to document the autopsy. Besides, nowadays, smartphones and tablets equipped with a LiDAR sensor make it extremely easy to elaborate a 3D model directly in the autopsy room. Herein, a quality and trustworthiness evaluation of 3D models obtained during ten autopsies is made comparing 3D models and conventional autopsy photographic records. Three-dimensional models were realistic and accurate and allowed precise measurements. The review of the autoptic report was facilitated by the 3D model. Conclusions: The LiDAR sensor and 3D models have been demonstrated to be a valid tool to introduce some kind of reproducibility into the autoptic practice.

## 1. Introduction

Autopsy is a complex and unrepeatable procedure. Therefore, it is essential to document all the procedural steps and findings to achieve legal significance. Traditionally, the forensic pathologist records the autoptic procedure with photographs, and this method is still widely used. However, two-dimensional (2D) images are not always adequate to record forensic practice because they can lead to distortions and misinterpretations, for example, in cases of skin and bone lesions [1]. When discussing a case in court with the purpose of justice, it is fundamental to rely on trustworthy images. In the last years, construct three-dimensional (3D) scanning has been introduced into this field [2,3]. One of the first methods applied was photogrammetry [4,5]. More recently, 3D surface extraction from computer tomography (CT) data and 3D scanning have also been used [6,7]. However, this method has some limits correlated to variability and the need for photographic skills [8]. A light detection and ranging (LiDAR) sensor is a remote sensing device that uses an infrared light pulsed laser to calculate the distance between two points [9]. Those data could then be combined with photographic information and used to construct 3D models of objects and/or surfaces. It is widely used in the engineering and construction field, as well as in many other disciplines [10]. The combination between the LiDAR scanner and the smartphone camera has made it possible to easily obtain 3D images of any objects, with a precise definition of their shape and surface. Before LiDAR, 3D documentation was time consuming and expensive [11]. Besides, usually it requires specific skills, for example, good photographic expertise or specific professional training, particularly in the case of CT 3D reconstruction [11,12]. Moreover, the equipment required for 3D documentation is not present in every facility (e.g., the 3D system “VirtoScan-on-Rails”, used by Kottner et al.) [13]. Several studies have demonstrated that 3D models could be a valid tool in forensic practice, in addition to other indispensable analyses (i.e., histology, toxicology, etc.) [14]. Three-dimensional documentation assists in findings visualization and display. Furthermore, it helps in events reconstruction. Additional use of 3D models is for medical education. Tóth et al. demonstrated that 3D reconstructions could be a supplementary tool in medical student training and preparation [15]. In their study, student satisfaction was higher with photogrammetry images than other education methods. Three-dimensional data have also been used to create 3D-printed organs [16,17].

In this work, the LiDAR sensor is presented as a new tool that can help forensic pathologists record their autoptic findings with high-quality 3D images.

## 2. Materials and Methods

### 2.1. Subjects

In this study, 3D models of entire corpses, single body-areas, and/or single organs were obtained during ten forensic autopsies using the app TRNIO. Three-dimensional models were then processed by MeshLab. The subjects were six males and four females, the age range was 22–74 y.o. The causes of death were various. Demographic details about the subjects are provided in Table 1.

The cases were selected from the autopsy databases of the Institutes of Legal Medicine of the University of Pisa and “Sapienza” University of Rome. All corpses were autopsied between 1 October 2021 and 31 October 2021. Autopsies previously performed were not included because we did not have the LiDAR-equipped device before that time. In all cases, histological analysis of tissues sampled during the autopsy was performed. Toxicological analysis, performed on peripheral blood, was negative for all cases. Table 2 shows the main autoptic (macroscopic and histological) findings of the ten cases included in this study, as well as the causes of death.

### 2.2. Equipment, Recording Method, and 3D Model Processing

Each autopsy procedural step was documented with a conventional camera and a device equipped with a LiDAR sensor. Therefore, we obtained a complete conventional autopsy photographic record and 3D autopsy record. Any peculiar finding (i.e., external lesions, organ alterations) was specifically documented.

A device equipped with a camera and a LiDAR sensor is required, apart from the conventional autopsy equipment. It is possible to use a tablet (iPad Pro) or a smartphone (iPhone 12 Pro, iPhone 12 Pro Max, iPhone 13 Pro, and iPhone 13 Pro Max); the prices of these devices vary from USD 999 to USD 1299. We used the iPhone 12 Pro, which is produced by Apple. According to the technical specifications provided by the manufacturer, it is sized at “146.7 mm × 71.5 mm”, weighs 189 g (6.66 ounces), and has three camera systems: Ultra-Wide (ƒ/2.4 aperture and 120° field of view), Wide (ƒ/1.6 aperture), and Telephoto (ƒ/2.0 aperture) cameras. Additional information could be found at the manufacturer’s website [18]. Unfortunately, the manufacturer does not provide further specifications of the LiDAR sensor integrated into the device.

**Figure 1:**
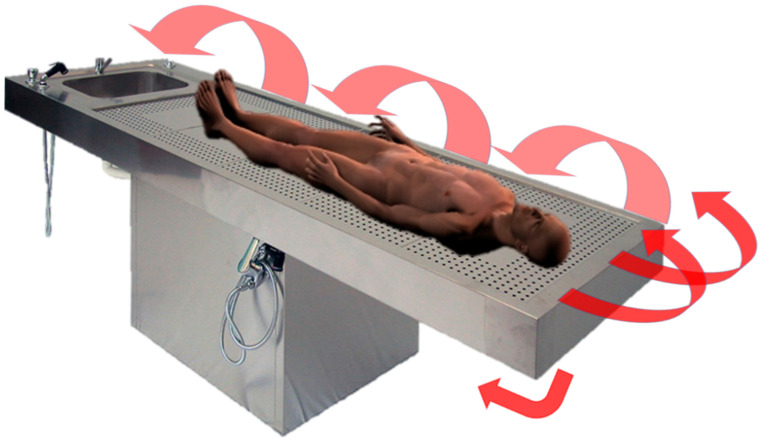
LiDAR 3D reconstruction of a corpse. The corpse was placed on a traditional autoptic steel table. The 3D model was created by capturing the surface data at first horizontally, and then the device was slowly moved to obtain surface data from various degrees. The arrows show how the camera of the device should be pointed, moved, and rotated to allow for capturing all the surfaces. When finished, the corpse should be placed in the prone position and the LiDAR scanning should be repeated.

**Figure 2:**
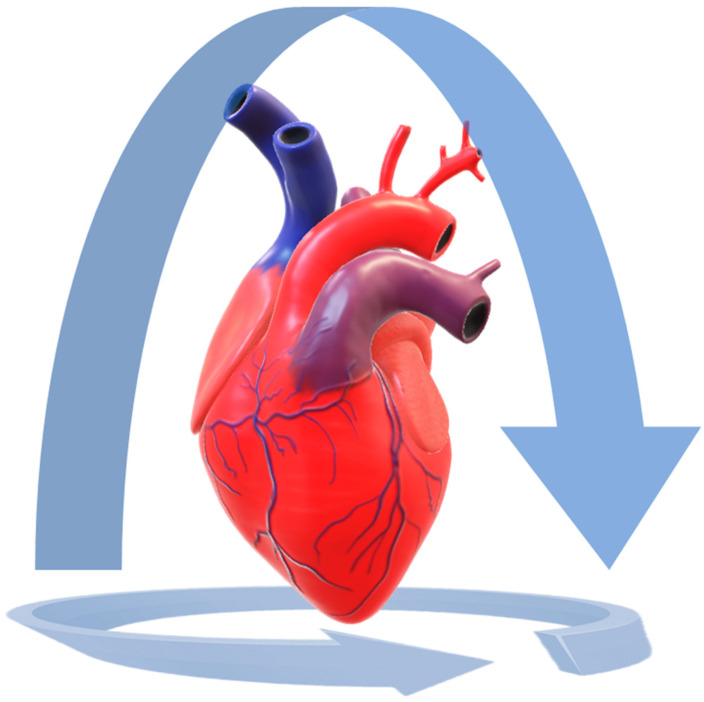
LiDAR 3D reconstruction of a heart. The heart was placed on a white plastic table, lying on its posterior surface. The 3D model was created by capturing the surface data from the anterior surface, then the camera was slowly moved in order to perform a complete rotation around the organ and to capture all the expose surfaces. Then the camera was moved to record the cardiac base. In this way, the heart was completely captured. The procedure should be repeated with the heart lying on its anterior surface.

A disposable plastic film cover could be used to protect the device if needed. No specific environmental conditions are required. The device could be used to capture the entire corpse or single organs/body areas. The camera should be pointed to the organ/body perpendicularly and then slowly moved to record all the surfaces of interest, as shown in Figure 1 and Figure 2.

This procedure takes only a few seconds, depending on the dimension of the surface of interest (for example, to record the entire body takes about 60–120 s). For better-quality images, the bearing surface should be flat and smooth. A polished steel table, as classically used for autopsy, is suitable for the purpose.

Two applications could be used to acquire data and then elaborate 3D images: TRNIO or 3D Scanner. Both applications are adequate for the purpose. 3D Scanner is free, TRNIO costs USD 4.99. The 3D reconstruction takes about five to ten minutes to be elaborated by the application.

It is possible to process the 3D models by MeshLab, an open-source tool for the visualization and processing of 3D models. MeshLab also provides a specific tool to measure any length of interest directly on the 3D model. The operator must select two points on the surface of interest (for example, two different lesions or the extremities of the same lesion), then the application provides the length between them in a few seconds. Besides, MeshLab could be used to process raw 3D digitization data for preparing models for 3D printing. MeshLab is freely available for all the major platforms (Windows, MacOS, Linux) [19].

### 2.3. Quality and Trustworthiness Evaluation

To evaluate the trustworthiness of 3D models, a comparison with conventional photography was then done, comparing:

− Conventional autopsy photographic records vs. 3D reconstruction: qualitative evaluation by consensus.
− Body/lesion measurement attained during the autoptic examination vs. body/lesion measurement attained from the 3D model. When a discrepancy was noticed, re-measurements were obtained.
− Lesion description and autopsy revaluation from conventional autopsy photograph records vs. from 3D image review: qualitative evaluation was performed by two forensic pathologists who did not attend the autopsy and did not have knowledge about the cases.

### 2.4. 3D Printing

MeshLab was also used to process a heart 3D model for 3D printing. The model was then 3D printed.

## 3. Results

**Figure 3:**
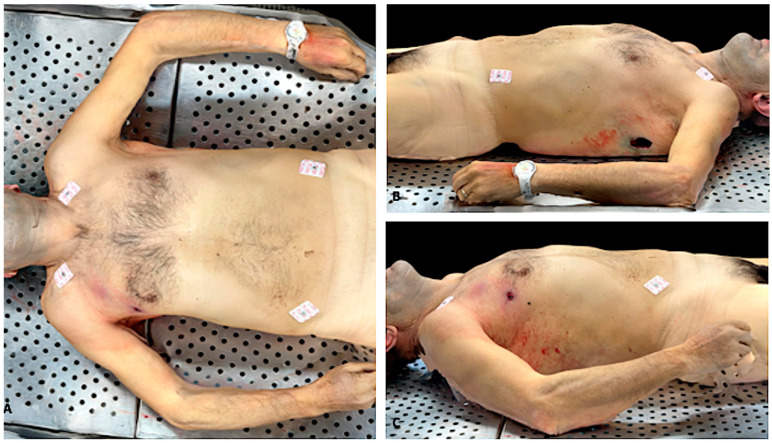
3D reconstruction of a corpse’s trunk and upper limbs (case 1). (**A**) gunshot wound is evident on both sides of the trunk (entrance on the right, exit on the left). (**B**) The reconstruction is realistic, accurate, and detailed. (**C**) Details that could allow personal recognition have been censored.

In all the cases, 3D models met our quality expectations. Three-dimensional images, compared to conventional autopsy photographic records, were realistic and accurate, the color rendering was truthful. Three-dimensional reconstruction was a valid method in recording autoptic findings. In Figure 3 and Video S1 (in Supplementary Materials), an example of 3D reconstruction is provided.

**Figure 4:**
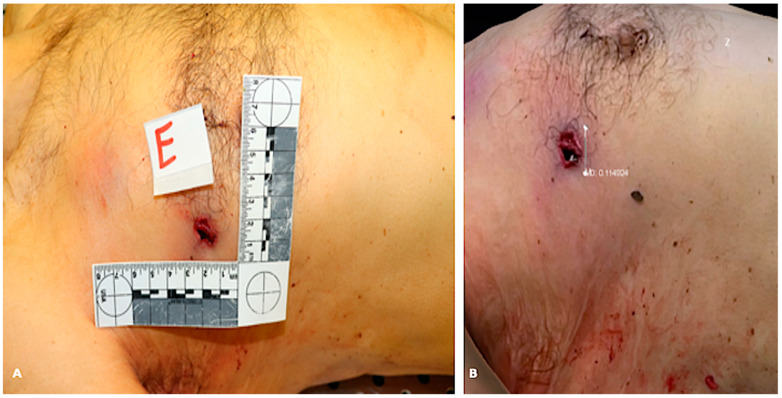
Comparison between 2D picture and 3D reconstruction of a corpse’s trunk (case 1). (**A**) shows the 2D picture with a ruler next to the gunshot wound (unit of measurement: centimeter). (**B**) shows the measurement of the gunshot wound diameter obtained from the 3D model (unit of measurement: decimeter). In this case, the initial “hand” measurement of the diameter of the lesion was 1.3 cm. We immediately checked it on the 3D model, which provided 1.14 cm, as shown in Figure 1B. We then re-measured the lesion and confirmed the true measure was the one provided by the 3D model.

The measurement of lesions or body parameters (i.e., body length) assessed on 3D models were trustworthy, even more so than the measurement obtained during the autopsy. In fact, in two cases we noticed a discrepancy between the “hand” measurement and the digital measurement, but when we re-measured the body/lesion of interest, we found out the reason was an operator inaccuracy, instead of LiDAR imprecision. Figure 4 shows an example of the comparison between traditional measurement and 3D model measurement.

**Figure 5:**
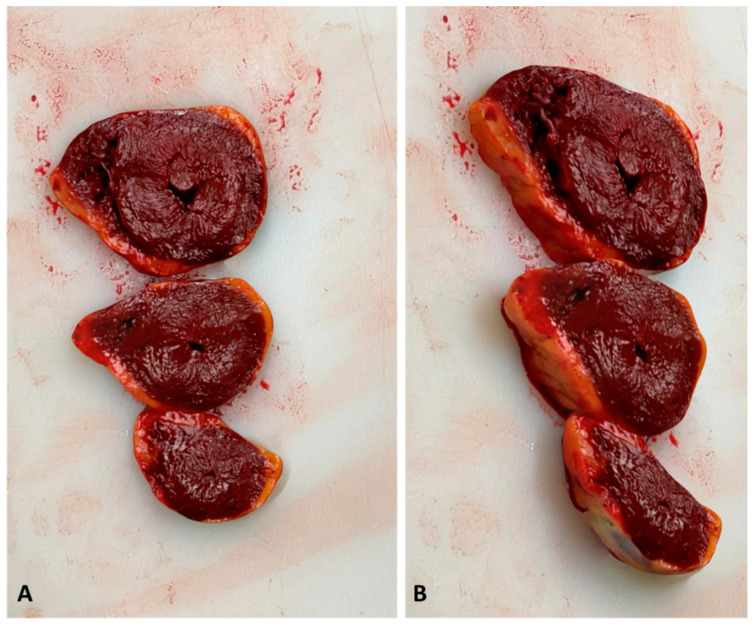
Three-dimensional reconstruction of the heart slides performed at the macroscopic examination of the heart (case 3). (**A**) shows a perpendicular view of the 3D model. (**B**) shows a lateral view. The myocardial wall is precisely measurable in the 3D model, sustaining the hypothesis of myocardial hypertrophy. In this case, the histologic examination confirmed the diagnosis.

Besides, when reviewing the autoptic procedure, the 3D model allows for more precise measurements than traditional images, because there is no 2D-induced distortion. We asked two forensic pathologists, who did not attend the autopsy and did not have knowledge about the cases, to re-evaluate both the conventional autopsy photograph record and the LiDAR reconstructions, in order to assess if 3D models could be used to audit forensic practice. The experts conveyed that external examination of the body and lesion measurements were accurate when performed on 3D reconstruction. In cases 2, 9, and 10 (see Table 2), the cause of death was fatal myocardial ischemia due to acute coronary artery disease. In these cases, the 3D reconstructions allowed the reviewer to measure the diameter of the coronary artery and to evaluate the percentage of lumen obstruction. In case 3, left ventricular hypertrophy was seen at the macroscopic examination of the heart. The reviewer easily suspected this cardiac alteration by reviewing the 3D reconstruction of the heart slides (Figure 5).

**Figure 6:**
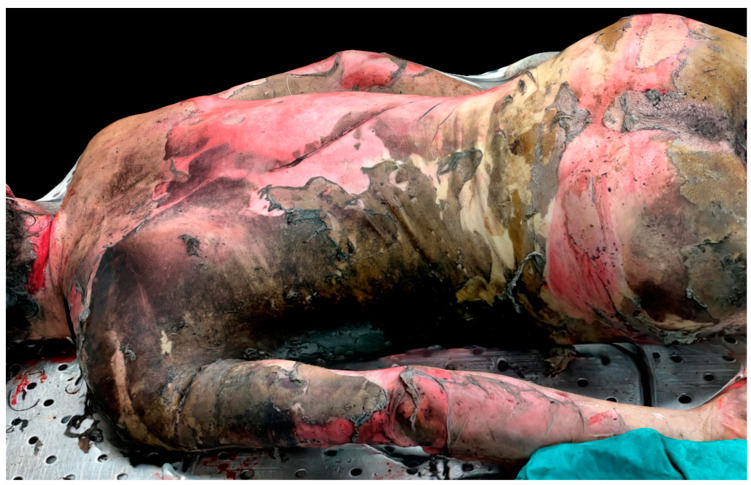
Three-dimesnional reconstruction of a corpse burned >40% of the body surface (case 4).

Furthermore, 3D images allowed us to evaluate the degree of depth of some lesions. This was obviously not possible with traditional pictures. In cases of complex traumatic lesion patterns, the audit of the autoptic process was facilitated by LiDAR reconstruction, because 3D images better showed the anatomical relationships between body structures (for example, the description of skull base fractures in case 7 and multiple costal fractures in case 8). Figure 6 shows the 3D reconstruction of a >40% burned body (case 4).

**Figure 7:**
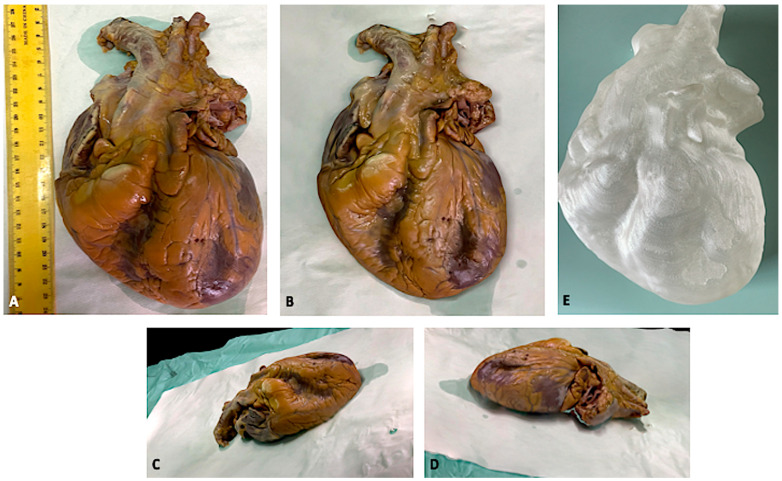
3D model of a formalin-fixed heart. (**A**) shows the traditional picture. (**B**–**D**) show the 3D reconstruction. (**E**) shows the 3D-printed heart.

As a supplementary activity, we also 3D printed the model of a heart. The reproduction was very accurate, as can be seen in Figure 7. Video S2 shows the heart 3D model.

## 4. Discussion

In our study, 3D models applied to the autoptic examination met the expectations and demonstrated trustworthiness in terms of quality and measurement accuracy. This technique preserves information in all three dimensions and so it is very useful in cases of traumatic lesions. Furthermore, measurements performed on the 3D reconstruction allowed us to avoid operator-related errors, not only associated with measurement imprecision but also in case the operator missed measuring or describing some finding during the autoptic examination.

Autopsy intrinsically destructs the object of its analysis, the corpse. Besides, putrefactive processes irreversibly altered the cadaver, and so, after a certain amount of time, exhuming and re-examining the body could be pointless. Photography allows one to maintain archival data and to audit the forensic practice. However, the quality and reliability of the photographic record and the autopsy report are not always guaranteed [20]. Technical requirements and good photographic skills are indispensable to perform a good photographic report, minimizing distortions and misinterpretations [21,22].

The introduction of radiological examinations into the forensic practice provided another way to record the pathological findings [23,24,25,26]. Nevertheless, post-mortem imaging entails considerable expenditure, both for the corpse transportation to the radiology facility and for the exam itself, and to perform those examinations is also time consuming. In the last years, 3D reconstruction has supplemented medico-legal activities, first of all in crime scene reconstruction [27,28,29]. Forensic pathologists also benefit from 3D surface documentation, but usually complex, expensive, and/or unwieldy instrumentations are required [1,30,31]. The introduction of photogrammetry has made 3D documentation easier than in the past since the required equipment consists basically of a camera and a computer [2,3,32,33,34,35]. However, this method has some limits. At first, the variability correlated to the type of camera [5]. Formerly, photographic skills are still necessary because the computer application needs good traditional photographs to create the 3D model. Besides, it requires a two-steps procedure: taking pictures during the autopsy and then, in a second moment, computer elaboration. It means that it is not possible to directly evaluate the 3D reconstruction quality in the autopsy room, so you cannot re-take a picture if you later discover it is not adequate.

Recently, LiDAR technology has met the mobile and tablet industry and nowadays smartphones/tablets with a LiDAR sensor next to the traditional camera have been placed on the market. This makes it extremely manageable to obtain 3D models of surfaces: you just need to point the camera at the object of interest (the body, the organ, the lesion) and slightly move, in order to create a complete reconstruction. No peculiar abilities or skills are required, and an everyday device is used for the purpose. TRNIO and 3D Scanner take from five to ten minutes to create the 3D model, so it is possible to verify the reconstruction directly in the autopsy room and to re-take it if necessary. In addition, this method is quite cheap if compared with other diagnostics examinations. The mobiles and tablets with a LiDAR sensor integrated cost about USD 1000, while other imaging machines cost thousands of dollars.

It is foreseeable that 3D models and 3D printing presentations in court would make medico-legal dissertations more effective and understandable. Besides, 3D printing could be useful in crime scene and traumatic dynamic reconstruction.

The introduction of this technology into the autoptic practice would also provide a new tool to review the autoptic findings. Unfortunately, the autopsy is not always followed by a good-quality report [36,37]. Sometimes, it could be necessary to audit the autopsy report, for example, when the process proceeds among the levels of justice. Three-dimensional models would guarantee a new form of reproducibility to the autopsy practice.

## 5. Conclusions

The autopsy is an unrepeatable procedure, but the autopsy report and the autopsy photographic record do not always meet the demanded standard of quality. In the last years, photogrammetry (3D images elaborated by photographs of the autoptic procedure) has been introduced into forensic practice. However, this technology still presents some limits.

The recent arrival on the scene of smartphones and tablets equipped with LiDAR sensors, in addition to the traditional camera, allows us to use this new technology in the autopsy room. Creating a 3D model of corpses, organs, and lesions has been made extremely easy and quick. In this study, 3D images obtained during autopsies were accurate and precise and have been demonstrated to be a valid tool in auditing and reviewing autoptic procedures.

Furthermore, displaying 3D models and 3D printings in court as evidence would probably improve the effectiveness and understandability of medico-legal dissertation. In conclusion, LiDAR technology could be a valid instrument that helps to introduce some kind of reproducibility into the autoptic practice.

## Figures and Tables

**Table 1:** Brief description of the subjects included in this work.

Case Number	Sex	Age (y.o.)
1	M	71
2	M	54
3	F	45
4	M	63
5	F	81
6	M	35
7	F	22
8	F	55
9	M	58
10	M	74

**Table 2:** Autoptic findings and causes of death of the ten cases included in this study.

Case Number	Macroscopic Findings	Microscopic Findings	Cause of Death
1	Entrance gunshot wound in the right side of the trunk, exit gunshot wound in the left side of the trunk	Hemorrhagic infiltration of soft tissues near the gunshot wounds	Gunshot
2	Coronary artery disease (atheromatous plaque)	Myocardial ischemia	Cardiovascular disease
3	Left ventricular hypertrophy	Diffuse myocardial interstitial fibrosis, myocardial hypertrophy	Cardiovascular disease
4	Diffuse burn lesions in various degrees, soot in the airways	Soot deposition in the medium and small airways’ mucosa, intraepidermal and subepidermal separation alongside coagulation necrosis in the skin	Fire burn > 40% body surface
5	Entrance gunshot wound in the oral cavity, several skull fracture	Hemorrhagic infiltration of soft tissues near the gunshot wound	Gunshot
6	Both ventricles dilation	Long and thin myocytes, interstitial fibrosis	Cardiovascular disease
7	Skull base fractures, lower limbs fractures, intracranial hemorrhage, and cerebral lacerations	Subarachnoid hemorrhage, hemorrhagic infiltration of soft tissues	Traffic accident
8	Multiple costal fractures, upper limbs fractures, multiple excoriations, heart lacerations, lungs ecchymoses	Hemorrhagic infiltration of soft tissues	Traffic accident
9	Coronary artery disease (atheromatous plaque)	Myocardial ischemia	Cardiovascular disease
10	Coronary artery disease (atheromatous plaque)	Myocardial ischemia	Cardiovascular disease

## Supplementary Materials

The following supporting information can be downloaded at: https://www.mdpi.com/article/10.3390/bios12020132/s1, Video S1. 3D reconstruction of a corpse’s trunk and upper limbs (case 1). Details that could allow personal recognition have been censored. Video S2. 3D model of a formalin-fixed heart.
